# Much Ado About Nothing: The Mental Representation of Omissive Relations

**DOI:** 10.3389/fpsyg.2020.609658

**Published:** 2021-02-03

**Authors:** Sangeet Khemlani, Paul Bello, Gordon Briggs, Hillary Harner, Christina Wasylyshyn

**Affiliations:** Navy Center for Applied Research in Artificial Intelligence, US Naval Research Laboratory, Washington, DC, United States

**Keywords:** omissions, absences, causal reasoning, mental models, negative possibilities

## Abstract

When the absence of an event causes some outcome, it is an instance of omissive causation. For instance, not eating lunch may cause you to be hungry. Recent psychological proposals concur that the mind represents causal relations, including omissive causal relations, through mental simulation, but they disagree on the form of that simulation. One theory states that people represent omissive causes as force vectors; another states that omissions are representations of contrasting counterfactual simulations; a third argues that people think about omissions by representing sets of iconic possibilities – mental models – in a piecemeal fashion. In this paper, we tease apart the empirical predictions of the three theories and describe experiments that run counter to two of them. Experiments 1 and 2 show that reasoners can infer temporal relations from omissive causes – a pattern that contravenes the force theory. Experiment 3 asked participants to list the possibilities consistent with an omissive cause – it found that they tended to list particular privileged possibilities first, most often, and faster than alternative possibilities. The pattern is consistent with the model theory, but inconsistent with the contrast hypothesis. We marshal the evidence and explain why it helps to solve a long-standing debate about how the mind represents omissions.

## Introduction

Omissions are events that do not occur – for instance, an otherwise chipper coworker might fail to greet you in the morning. The coworker’s omission might indicate stress, and so people can use omissions to diagnose other states of affairs. And omissions can participate in causal relations, too: the absence of a particular action can cause some state of affairs to come about, such as when a taxpayer’s failure to file her taxes leads to fines. As the example suggests, the costs of a failure to act can have grave personal and legal consequences (Ferrara, 2013). Despite how common omissions are in daily life, philosophers and psychologists have difficulty characterizing them in the same way as they characterize orthodox (i.e., non-omissive) causes. An orthodox causal relation, as in (1):

1. Filing her taxes caused a taxpayer to collect a refund.

concerns a relation between two events (i.e., *filing her taxes* and *collecting a refund*). But omissions are non-events, and it is unclear how a non-event can be an argument to a causal relation. It may be compelling to think of omissions in omissive causes as nothing whatsoever. The idea is a prominent view among many philosophers (Hommen, 2014). For instance, Moore (2009, p. 444) argues that when people assert omissive causal statements akin to *A not happening caused B*, “[they] are saying that there was no instance of some type of action *A* at [time point] *t* when there is an omission to *A* at *t.*” Likewise, Sartorio (2009, p. 513) argues that “it’s hard to count omissions as [causal] actions, for omissions don’t appear to have specific spatiotemporal locations, intrinsic properties, etc.” Other theorists similarly defend the idea that omissions are non-entities: they have no metaphysical substance. They do not convey facts, truths, or presuppositions; they are not states of affairs or possibilities; they are not un-instantiated actions; and they are not features of space-time regions. They’re just…*nothing* (see Clarke, 2014, p. 38 et seq.; cf. Nelkin and Rickless, 2015).

The trouble for philosophers is that causal relations are about something, not nothing. For Hume (1748/1988), causation described an observed regularity between one event – a cause – and another event – its effect. As he wrote (p. 115): “We may define a cause to be an object followed by another, and where all the objects, similar to the first, are followed by objects similar to the second.” But it is impossible to observe a non-event, such as the failure to pay taxes. The metaphysics of omissions is so problematic that some philosophers even deny that omissive causation is a meaningful concept (e.g., Dowe, 2001; Beebee, 2004; Hall, 2004; see Pundik, 2007, for a review).

Cognitive scientists cannot reject the psychological reality of omissive causation, because people have no difficulty understanding causal statements that describe omissions, and indeed, they can draw systematic conclusions from omissive relations (Wolff et al., 2010; Stephan et al., 2017; Khemlani et al., 2018b). For instance, reasoners can draw transitive deductions from omissive causes. Briggs and Khemlani (2019) presented participants with arguments of the following structure:

2. Not having X causes Y.   [an omissive causal relation]

Having Y causes condition Z.   [an orthodox causal relation]

What, if anything, follows?

where *X*, *Y*, and *Z* were replaced by fictitious medical conditions. The majority of participants (66%) spontaneously generated the conclusion that *not having X causes Z*. Hence, they inferred an omissive cause. The result replicates a similar pattern found in studies by Wolff and Barbey (2015), and it suggests, first, that people’s inferences about omissive causes are predictable, and second, that people’s mental representations of omissive causes are productive: they allow reasoners to draw novel conclusions. Hence, it is unlikely that people represent omissions as nothing whatsoever – they must represent the omitted event in some capacity. How they represent omissions remains controversial; the earliest psychological proposal argues that people represent omissive causation as simulated arrangements of forces (Wolff et al., 2010). Another idea asserts that reasoners represent counterfactual contrasts (Stephan et al., 2017): one representation for the omitted event, and another representation for what could have been, i.e., the event itself. And yet others have argued that they represent one or more possibilities that simulate the scenario (Khemlani et al., 2018b); they represent omissions as negated possibilities.

Our goal in this paper is to survey and test recent theoretical proposals about the mental representation of omissive causation. In the next section, we review principal phenomena in causal reasoning that any reasonable theory should explain. In a subsequent section, we describe in depth the proposals outlined above:

1. that omissions are simulations of forces;
2. that omissions are contrasting simulations;
3. that omissions are simulations of possibilities.

We spell out their various predictions. We then describe four experiments that put the proposals to test. Two experiments rule out the view that omissions are forces. A third study rules out the idea that omissions are contrasts. And each study corroborates the hypothesis that people simulate omissions as negated possibilities. We conclude by discussing why the study of omissions should be central to theories of causation, and we review how the present results can inform theoretical and computational models of general-purpose causal reasoning.

## Principal Phenomena in Causal Reasoning

Omissions have psychological import because they affect the conclusions people draw from causal statements (Wolff et al., 2010). People appear to interpret, represent, and reason about omissive relations similarly to how they reason about orthodox causation (see, e.g., Waldmann, 2017 for a comprehensive review on research into orthodox causation). There are at least three principal phenomena that characterize reasoning about omissions. First, reasoners distinguish between different kinds of relations between two events, i.e., they reason differently about causes, enabling conditions, and preventions (Cummins, 1995; Goldvarg and Johnson-Laird, 2001; Wolff, 2007; Sloman et al., 2009; Johnson-Laird and Khemlani, 2017). Hence, in the sentences describing omissions below, the italicized causal verbs cannot be swapped with one another:

3 a. Not exposing a flower to light *causes* it to die.
b. Not plucking a flower *enables* it to bloom.
c. Not pollinating a flower *prevents* it from making seeds.

That is (3b) describes an enabling condition between *not plucking a flower* and *blooming*, and no other relation is appropriate. As a result, the following sentences are incoherent:

4 a. # Not exposing a flower to light *prevents* it from dying.
b. # Not plucking a flower *causes* it to bloom.
c. # Not pollinating a flower *enables* it to make seeds.

where # denotes incoherence. Similar distinctions hold for orthodox causation: it is acceptable to say, for instance, “unlocking a door enables it to open” and “pushing a door causes it to open,” but it is incoherent to say “pushing a door enables it to open.”

Second, causal relations are inherently temporal. Hume (1739/1978) argued that causes precede their effects, and many events in everyday causal reasoning abide by Hume’s proposal. Hume rejected the idea that causes and effects can occur simultaneously, but contemporary science condones simultaneous causation, e.g., the gravitational pull of the sun causes the earth’s orbit (see also Kant, 1781/1934; Taylor, 1966). Hence a reasonable constraint is that effects do not precede their causes (Tversky and Kahneman, 1980; Bullock et al., 1982). Reasoners appear willing to draw temporal conclusions from orthodox causal assertions (Bechlivanidis and Lagnado, 2016), and in some cases, temporal orders serve as a cue for causality (Burns and McCormack, 2009; Bramley et al., 2014; McCormack et al., 2015). For instance, suppose a parent is given the following reason for why her son failed a particular class:

5. Cheating on a test caused him to fail.

The parent can sensibly interpret the temporal order of events: her child cheated *first* then failed *afterward*. The inference seems trivial, and any reasonable account of causal reasoning should be able to explain it.

Drawing temporal conclusions from omissive causes can seem more controversial, particularly given the aforementioned philosophical concerns over omissions. Consider the following alternative reason for the son’s failure:

6. Not doing his homework caused him to fail.

If omissions are nothing, as many philosophers argue, then they have no place in space and time. Such philosophers should argue that it does not make sense to infer any temporal relation between the events in (6) – i.e., it does not make sense to infer that the student didn’t do his homework before his grade fell – because his failure to do homework did not occur in the context before or after any other event. It simply did not occur. But no studies have ever examined what kinds of temporal inferences people draw from (6), or if they draw any at all. As we will show below, almost all reasoners draw temporal conclusions from such statements.

Third, causal relations allow reasoners to consider alternative possibilities of how the relevant events could have transpired, or else, how the events might transpire. For instance, (5) above describes a fact, but it also appears to imply the following counterfactual conditional: *if the son hadn’t cheated, he might not have failed.* The conditional is consistent with two separate counterfactual possibilities:

7 a. The son didn’t cheat and he didn’t fail.
b. The son didn’t cheat, but he failed for some other reason.

Counterfactuals are relevant for retrospective causal relations, such as the one described in (5). But prospective causal relations can describe future scenarios, as in the following:

8. A spike in unemployment will cause wages to fall.

The statement is consistent with the following possibilities:

9 a. Unemployment spikes, and wages fall.
b. Unemployment doesn’t spike, and wages don’t fall.
c. Unemployment doesn’t spike, but wages fall for some other reason.

The causal relation described in (8) does not describe an inevitability: it permits reasoners to consider multiple possibilities in which the future might manifest. The same argument could be made for omissive relations concerning future events:

10. Not paying a parking meter appropriately will cause you to get fined.

The statement in (10) seems true even if you have already paid the meter, and so it is consistent with multiple possibilities.

In sum, psychological theories of omissive causation should explain, at a minimum: how people distinguish between omissive causes and omissive enabling conditions, how they infer temporal relations from causal ones, and how people consider alternative possibilities consistent with the causal relations.

## Cognitive Accounts of Omissive Causation

Philosophers are concerned by the metaphysics of omissions (Dowe, 2001; Beebee, 2004; Hall, 2004; Pundik, 2007), which is why some have argued that omissions are nothing whatsoever (Moore, 2009; Sartorio, 2009; Clarke, 2014; Hommen, 2014). Psychologists cannot endorse such a view: omissions can be articulated, and psychologists take for granted that the human mind must represent anything that can be articulated. But they have yet to concur on how the mind represents omissions. We describe three recent proposals below, and illustrate the predictions that distinguish them.

### Omissions as Forces

A prominent theory of omissive relations comes from Wolff et al. (2010), who argue that people represent omissions as a set of interacting forces. The force theory posits that individuals represent the direction and magnitude of a causal *force*, i.e., a tendency to direct an entity to a particular outcome (Wolff and Barbey, 2015). The theory was inspired by Talmy’s (1988) force dynamics theory, in which forces are represented as vectors (see also De Mulder, 2010, for a more recent analysis). Under Wolff et al.’s (2010) account, events are assigned forces in relation to one another. Hence, (5) above can be represented by two separate forces: one force describes the child’s school performance in the absence of cheating, which moves in the direction of passing the course. And another force describes the effect of cheating on school performance, such that cheating directs performance toward failure. An advantage of the theory is that it provides a way to distinguish between causes, enabling conditions, and preventions. Preventions, for instance, are similar to causes, i.e., they concern situations in which a force redirects the trajectory of a particular outcome. The theory can accordingly explain why the situation described in (5) is equivalent to the following:

11. Cheating prevented the child from passing.

Omissive causes seemed, at first, to challenge the force theory: the absence of an event should be equivalent to the absence of a force, and so, as critics of the theory argued, the force theory is unable to explain omissive causation – though nobody had developed an adequate alternative account of omissive causation at the time. Wolff et al. (2010, p. 193) responded to the criticism by proposing the first such theory. They argued that omissions are equivalent to “double preventions,” i.e., scenarios in which one entity prevents another entity from preventing an outcome:

“…absences are causal when the removal or non-realization of an anticipated force leads to an effect… consider a situation in which a car is held off the ground by a jack. A man pushes the jack aside—removing the force holding up the car—and the car falls to the ground. This situation instantiates a type of causation by omission, as indicated by the acceptability of the description ‘The lack of a jack caused the car to fall to the ground.’ …[The force theory proposes] that causation by omission is always embedded within a double prevention. In double preventions, the second entity [e.g., the jack] is removed, and so the relationship between the second and third entities [i.e., the jack and the car’s fall] concerns what happens to the third entity in the absence of the second entity.”

That is, the force theory interprets causation by omission, as in *the absence of A causes B*, as *X prevents A* and *A prevents B*. Philosophers have invoked double preventions to explain omissive causation (Collins, 2000; Hall, 2000, 2004; Dowe, 2001), but, unlike philosophical proposals, the force theory appeals to the vector calculus in mathematics to explain how two preventative forces can be composed to yield double preventions. The theory therefore provides a unified account of how people might interpret (5) and (6) above.

The force theory faces two overarching challenges. First, it appeals to double prevention to explain, not just omissive causes, but enabling conditions as well. It therefore predicts that people will often conflate omissive causes with omissive enabling conditions (“allowing” relations): “in the absence of clear knowledge of the magnitudes, double preventions will be most naturally described as ALLOW relations” (Wolff et al., 2010, p. 198). The authors present evidence for such conflations, but, as Khemlani et al. (2018b) show, reasoners are capable of distinguishing omissive causes from omissive enabling conditions (see also the preceding section).

Second, because the theory appeals to the vector calculus to explain how forces combine, it has no way of representing time. The vector calculus treats force vectors as atemporal – vectors encode only the direction and magnitude of a particular event. To make predictions about causal inferences, the theory composes an outcome vector from two different force vectors representing causal relations. Hence, the outcome vector cannot be used to represent the relative time at which any events took place, nor can it represent the way a cause can precede its outcome. In some cases, following Talmy (1988), force theorists describe forces as “affectors” that shift an entity toward an “end state” (see, e.g., Wolff and Thorstad, 2017). But despite the use of the temporal term “end,” the theory provides no mechanism for extracting temporal relations from force vectors. Computational implementations of the theory likewise do not yield representations of temporal order, and so, in general, the theory cannot explain how people draw temporal inferences from both orthodox and omissive causes. As we noted earlier, however, reasoners have no difficulty drawing temporal relations from orthodox causes (Bechlivanidis and Lagnado, 2016). No studies have examined whether they likewise draw temporal relations from omissive causes – such a result would undermine the force theory. We accordingly designed two experiments to test the matter.

### Omissions as Contrasts

A contrasting proposal, so to speak, concerns contrasts. When you think about the omissive cause in, say, (6) above, you consider two contrasting scenarios:

12 a. Not doing homework and failing. (not-A and B)
b. Doing homework and passing. (A and not-B)

Philosophers such as Bernstein (2014) have proposed that omissions denote a “non-actualized possibility,” as in (12b). Bernstein invokes the machinery of possible worlds to argue that omissions involve “counterpart relations” between actual omitted events and non-actualized contrasting events. A related idea by Schaffer (2005) is that omissions denote actualized alternative events, e.g., the event that occurred instead of the child doing homework, such as the child playing video games.

Neither of the two accounts were meant to be psychologically plausible, but a recent computational theory by Gerstenberg et al. (2015) and Stephan et al. (2017) bases a cognitive account of omissions on the idea that people represent an omission as a fact (the omission, as in 12a) and a counterfactual contrast (as in 12b). Gerstenberg and colleagues argue that counterfactual contrasts help explain the difference between causes, enabling conditions, and preventions (Gerstenberg et al., 2015; Gerstenberg and Tenenbaum, 2017). One clear advantage of the theory (particularly as outlined in Stephan et al., 2017) is that it can, in principle, explain how people draw temporal conclusions from causal relations. The account treats omissive causes as counterfactual contrasts in a physics engine, and physics engines contain veridical internal clocks, so they can explicitly represent points in time – hence, temporal order could be computed from the operations of the engine. It may be a challenge to ascertain how such operations map onto psychological constructs, since humans don’t possess a veridical clock. But, the theory nevertheless treats counterfactual simulations as inherently temporal. Evidence from the psychology of counterfactual reasoning suggests that reasoners are in principle capable of maintaining two separate possibilities (Byrne, 2005).

Stephan et al.’s (2017) proposal is compatible with two patterns of data: first, reasoners may interpret omissive causes as referring to two representations by default. If they sample one of the two contrasting possibilities (in accordance with the possibility sampling framework proposed by Phillips et al., 2019; see also Henne et al., 2019), they should tend to sample the alternative. Hence, they should show no bias toward the possibilities described in (12a) or (12b). Second, reasoners may understand an omissive cause by reference to an orthodox contrast. For example, when a reasoner interprets *not-A causes B*, they should sample *A and not-B* first, and *not-A and B* second. Recent work by Gerstenberg and Tenenbaum (2017) shows that people’s eye-movements appear to track and anticipate such orthodox contrasts.

Two patterns of data contravene the contrast theory: one is if people interpret the omissive possibility first and more often, and the orthodox contrast second and less often. This pattern would suggest that people represent omissive causes directly, and that their representation need not depend on a contrast. A second, less obvious pattern would also falsify the theory: if participants treat omissive causes as referring to three possibilities. The contrast theory proposes that people should only represent a single contrast. But, consider the example of the child’s failure above in (6); when interpreting the relation, some reasoners might consider a third possibility:

13. Doing homework and failing. (A and B)

In other words, reasoners might consider the possibility in which the child did his homework but failed anyway, such as a case in which he plagiarized his work. Below, we describe an experiment that tests for these patterns.

### Omissions as Models

A recent psychological theory of omissions assumes that reasoners represent omissive causes as mental simulations of sets of iconic possibilities (Khemlani et al., 2018b), i.e., models. Models are iconic insofar as their structures reflect the structures of what they represent (Peirce, 1931–1958, Vol. 4). Hence, a model of a spatial relation such as *the apple is to the left of the banana* is a representation in which a token that represents an apple is located to the left of a token that represents a banana, as in this diagram:

apple   banana

Inferences emerge from iconic representations (Goodwin and Johnson-Laird, 2005). For instance, reasoners can infer that the banana is to the right of the apple. Some concepts cannot be represented in an iconic way, and so the model theory allows for symbols to be integrated into models, such as the symbol denoting negation (Khemlani et al., 2012). Symbols cannot be integrated into, e.g., physics simulations like those used in the contrast theory.

The model theory proposes that people represent omissive causes as negated, temporally ordered possibilities (Khemlani et al., 2018b). For instance, the statement in (6) refers to three separate possibilities that can be depicted in the following diagram:

**¬ homework**     **failed**
   homework      ¬ failed
   homework      failed

The diagram is iconic such that it depicts a temporal order in which time moves from left to right. Each row of the diagram therefore represents a different temporally ordered possibility in which (6) could be true, and ‘¬’ denotes the symbol for negation. Hence, the first row depicts the possibility in which the student didn’t do his homework and his grade then fell; the second row depicts the possibility in which he did his homework and his grade didn’t fall (a counterfactual possibility; see Khemlani et al., 2018a); and the third row depicts the possibility in which he did his homework but his grade fell for some other reason (an alternative counterfactual). The causal relation in (6) is incompatible with the situation in which he didn’t do his homework and he passed (i.e., he didn’t fail):

¬ homework   ¬ failed

The model of (6) above does not directly represent that situation, since it represents only those possibilities that are consistent with the premise.

The theory explains the difference between omissive causes and omissive enabling conditions. Consider the following:

13. Not cheating enabled him to pass.

Enablers are distinct from causes, because nothing guarantees an enabled outcome to occur – just because he didn’t cheat doesn’t mean he passed. So, the models of (13) are:

**¬ cheating**     **passed**
¬ cheating      ¬ passed
   cheating      ¬ passed

Unlike the models of a causal relation, enabling conditions such as the one in (13) are compatible with the following situation:

¬ cheating      ¬ passed

and are incompatible with this situation:

cheating      passed

Recent experiments gave participants vignettes describing causal relations and enabling conditions, and found that they distinguish the two based on the possibilities as outlined above (Khemlani et al., 2018b).

One corollary of the model theory is that it is incompatible with the view that people consider contrasting possibilities by default. Its central tenet is that each model demands cognitive resources, and so it posits that people typically reason with just one possibility at a time – that is, the bolded possibilities in the sets of models for causes and enabling conditions above. Those possibilities denote *initial* models, i.e., privileged scenarios that come to mind first and foremost:

¬ homework   failed

An initial model can be scanned and combined with models of additional premises to make rapid inferences, but reasoners who rely on an initial model and not the full set of possibilities are vulnerable to systematic errors (see Khemlani and Johnson-Laird, 2017, for a review). Those who do construct the full set of possibilities tend to tax their working memory resources, so they should respond slower than when they rely on mental models alone.

Because a model of a causal relation concerns a temporally ordered possibility, the theory further predicts that people should draw temporal inferences from both orthodox and omissive causal relations. They should do so systematically, not haphazardly, i.e., they should infer from (6) that the student didn’t do his homework *before* he failed, but they shouldn’t infer that he didn’t do his homework after he failed, because the initial model of (6) is incompatible with such a possibility.

In sum, the three theories of the representation of omissions make different empirical predictions. Table 1 outlines them. Khemlani et al. (2018b) found evidence that corroborated prediction 1, and so the remainder of the paper therefore describes experiments that tested predictions 2, 3, and 4. Experiments 1 and 2 examined whether people make systematic temporal inferences from omissive causes (prediction 2). Experiment 3 tested whether people consider contrasting possibilities by default – as the contrast theory would predict – or whether people privilege certain possibilities over contrasts – as the model theory would predict (prediction 3). And Experiment 3 also tested how many possibilities people consider when reasoning about causation: the force theory predicts that people should consider one possibility, i.e., the combined vector of forces; the contrast theory predicts that people should consider two possibilities, i.e., an omission and its contrast; the model theory predicts that people should consider three possibilities, i.e., the initial model and its two alternatives.

**TABLE 1:** Predictions of the theoretical proposals of the representation of omissive causes as well as the datasets that test the various predictions.

Predictions	Theories of omissive causation			Relevant datasets
	
	Omissions are forces	Omissions are contrasts	Omissions are models	
1. People distinguish omissive causes, enablers, and preventions	No	Yes	Yes	Khemlani et al., 2018b
2. People make temporal inferences from omissive causes	No	Yes	Yes	Expts. 1 & 2
3. People consider contrasts by default	No	Yes	No	Expt. 3
4. People can consider *n* separate possibilities corresponding to omissive relations	1	2	3	Expt. 3

## Experiment 1

Experiment 1 tested the prediction that people draw temporal inferences from omissive and orthodox causal assertions. As Table 1 shows, the model theory and the contrast theory can account for such behavior, whereas the force theory has no mechanism to explain it. Participants were given a statement of the following schematic structure:

[Doing/not doing] A caused B.

Their task was to respond to a question of the following format:

Did [A/not A] occur before B? [cause-before-effect]

Half of the problems asked participants to evaluate temporal relations in which the causal event, i.e., event *A* (or *not A*) occurred before event *B*, and the other half of the problems presented participants with the events reversed:

Did B occur before [A/not A]? [cause-after-effect]

Both the model and the contrast theories predict that reasoners should respond “yes” to the first question, regardless of whether the events concerned an orthodox or an omissive cause. And they likewise predict that people should reject the second question.

### Method

*Participants.* 50 participants (mean age = 37.6 years; 31 males and 19 females) volunteered through the Amazon Mechanical Turk online platform (see Paolacci et al., 2010, for a review). 17 participants reported some formal logic or advanced mathematical training and the remaining reported no training. All participants were native English speakers.

*Design, procedure, and materials.* Participants carried out the experiment on a computer screen. The study was designed in psiTurk (Gureckis et al., 2015). After reading instructions, participants carried out a practice problem and then completed 12 experimental problems. Problems consisted of a causal premise and a question concerning a temporal relation. The events in the causal premise concerned the casting of magical spells (causes) and their fictitious effects. Half the problems concerned omissive causation by describing what occurred when a particular spell wasn’t cast (e.g., “Not casting allimon.”); and the other half concerned orthodox causation by describing spells that were cast (e.g., “Casting allimon…”). The effects of the spells concerned fictitious diseases that afflicted a particular individual (e.g., “…caused Peter to have kandersa disease.”). After reading the causal premise, participants were asked a question about a temporal relation. The format of the question depended on whether the causal premise described omissive or orthodox causation. For instance, if the premise described orthodox causation, the temporal relation concerned a cause and its effect, e.g.,

Did casting allimon occur before Peter’s kandersa disease occurred?

And if the premise described omissive causation, the temporal relation described a non-event and its effect:

Did not casting allimon occur before Peter’s kandersa disease occurred?

On half of the problems, the question described a relation in which the cause occurred before the effect, and on the other half, the order was reversed. Participants responded by choosing one of three different options: “Yes,” “No,” and “Don’t know for sure.” The information for each problem was presented simultaneously, and participants could not continue without selecting one of the three options. The presentation order of the problems and the materials was randomized, as was the order of the three response options on the screen.

### Results and Discussion

**FIGURE 1:**
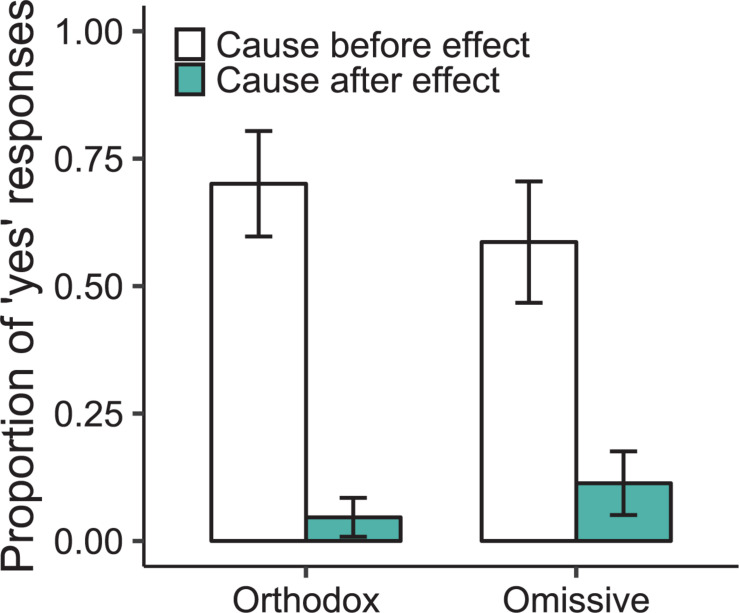
Proportions of “yes” responses in Experiment 1 as a function of whether the causal relation concerned an orthodox or an omissive cause and as a function of whether the temporal relation evaluated described a cause that occurred before or after the effect. The balance of responses in the study were either “No” or “Don’t know for sure.”

Figure 1 shows participants’ proportions of “yes” responses as a function of whether the inference concerned omissive or orthodox causation and as a function of whether participants evaluated the temporal order in which the causal event occurred before the effect or after it. Data were subjected to a generalized logistical mixed-model (GLMM) regression analysis using the *lme4* package in R (Bates et al., 2015) that utilized a maximal random-effects structure (following Barr et al., 2013) and treated the type of causation (orthodox vs. omissive) and the temporal order (cause-before-effect vs. effect-before-cause) as fixed effects. Participants’ percentages of “yes” responses did not differ as a function or whether the inference described an omissive or an orthodox causal relation (35 vs. 37%; *B* = *0.69*, SE = 0.56, *p* = 0.22). They responded “yes” more often to temporal relations when those relations described a cause that occurred before an effect rather than after (64 vs. 8%; *B* = 3.85, SE = 0.95, *p* < 0.0001). The interaction between the effect of the causal relation and the effect of the temporal relation was not reliable (*B* = 2.17, SE = 1.76, *p* = 0.22). Pairwise comparisons of estimated marginal means (conducted using the “emmeans” package in R; see Lenth, 2020) revealed that participants’ selections of “yes” responses to cause-before-effect relations occurred reliably higher than chance, both for orthodox causes (*B* = 4.536, SE = 0.95, *p* < 0.0001) and for omissive causes (*B* = 3.85, SE = 0.95, *p* = 0.0003).

Participants in Experiment 1 validated prediction 2 (see Table 1). Reasoners inferred temporal relations from causal statements for both orthodox and omissive causes, and they inferred only those temporal relations that matched the temporal order predicted by the use of either physics simulations or models. The absence of a reliable difference between orthodox and omissive causation lends further credence to the notion that the mental processes concerning omissive causes are similar to those concerning orthodox causes.

Experiment 1 was limited in that the task was evaluative, and so on each problem, participants were asked to infer a single temporal relation, namely *before*, which may have prevented them from considering alternative temporal relations. For example, participants often responded “Yes” to the following question about omissions:

Did not casting allimon occur before Peter’s kandersa disease occurred?

Their affirmation might allow that the omissive cause (“not casting allimon”) also occurred after Peter’s kandersa disease occurred, but the evaluative nature of the task prohibited any such analysis. Another limitation of the task is that participants may have misconstrued it as asking about possibility, not necessity, and so perhaps they affirmed the temporal relation because they considered it a viable possibility. Experiment 2 ruled out these concerns.

## Experiment 2

Experiment 2 was similar in design and execution to Experiment 1: the problems presented omissive or orthodox causal assertions paired with an assertion that described a potential temporal relation between events. However, the second assertion given to participants was incomplete, and their task was to fill in the blank. Half the problems took on the following general structure:

Suppose the following statement is true:

[Doing/not doing] A caused B.

Given the above statement, complete the following sentence:

[A/not A] occurred ________ B occurred. [cause-before-effect]

and the other half of the problems reversed the order of the events:

B occurred ________ [A/not A] occurred. [cause-after-effect]

Participants’ task was to choose among three different options to fill in the blank: “after,” “before,” and “and also.” The last option permitted them to be agnostic about when events or non-events occurred in relation to each other, and so participants could select them as most appropriate for omissive causal relations. Prediction 2 suggests, instead, that reasoners should select “before” or “after” depending on the order of events in the incomplete sentence, and that they should treat orthodox and omissive causal relations similarly.

### Method

*Participants.* 50 participants volunteered through the Amazon Mechanical Turk online platform (mean age = 39.8 years; 28 males and 22 females). 30 participants reported no formal logic or advanced mathematical training and the remaining reported introductory to advanced training in logic. All were native English speakers.

*Design, procedure, and materials.* Participants completed 1 practice problem and 12 experimental problems, and they acted as their own controls. Each problem consisted of a causal assertion and presented participants with an incomplete sentence. The experiment manipulated whether the first event concerned orthodox or omissive causation. It also manipulated the order of the events in the incomplete sentence: half the problems described the cause, a blank relation, and the effect; and the other half of the problems described: the effect, a blank relation, and the cause. The problems used the same materials as in Experiment 1 an example (omissive causal) problem was as follows:

Suppose the following statement is true:

Not casting allimon caused Peter to have kandersa disease.

Given the above statement, complete the following sentence:

Peter’s kandersa disease occurred ________ not casting allimon occurred.

Three separate response options (“before,” “after,” and “and also”) were presented as a dropdown menu to fill in the blank in the incomplete sentence. Participants were prevented from moving on to the next problem until they selected one of the three options. The presentation order of the problems was randomized, the contents of the problems were randomized, and the order in which the three response options appeared in the dropdown menu was randomized.

### Results and Discussion

An initial analysis examined participants’ tendency to select “after” or “before” as a function of the type of cause in the causal assertion. No reliable differences occurred in their tendency to select “before” as a function of whether the causal assertion in the problem concerned an omissive or an orthodox cause (43 vs. 47%; Wilcoxon test, *z* = 1.65, *p* = 0.09, Cliff’s = 0.11) and likewise for their tendency to select “after” (43 vs. 48%; Wilcoxon test, *z* = 1.09, *p* = 0.28, Cliff’s δ = 0.10). Follow-up GLMM analyses that utilized maximal random-effects structures likewise revealed no reliable difference between the tendency to select “before” (*B* = 0.84, *p* = 0.31) or “after” (*B* = −2.81, *p* = 0.15) as a function of whether the causal relation was orthodox or omissive. The result corroborates the model theory’s first prediction. In what follows, we pooled the data for orthodox and omissive causes except for one *post hoc* planned comparison.

**FIGURE 2:**
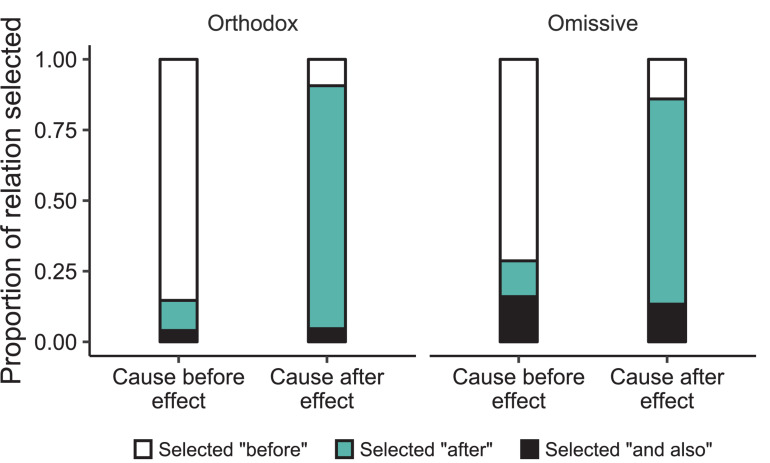
Proportions of participants’ selections of the three different types of relations in Experiment 2 as a function of whether the incomplete assertion described a cause that occurred before or after the effect for both orthodox and omissive causation.

Figure 2 shows participants’ tendency to select “before,” “after,” or “and also” responses as a function of the sequential order of the terms in the incomplete statement. Their tendencies to select each separate response were subjected to three different GLMM regressions. Participants selected “before” more often when the cause occurred before the effect than vice versa (78 vs. 12%; B = −5.19, SE = 1.38, *p* = 0.0002), and they selected “after” more often when the effect occurred before the cause than vice versa (79 vs. 12%; B = 4.39, SE = 0.84, *p* < 0.0001). Selections of “and also” responses did not differ as a function of the sequential order of events in the incomplete sentence (10 vs. 9%; B = −0.46, SE = 2.02, *p* = 0.82). No other fixed effects were reliable across the three regressions.

A *post hoc* planned comparison sought to test whether participants selected “and also” responses more often for omissive causes than orthodox causes (14 vs. 4%; B = 1.54, SE = 2.25, *p* = 0.49). Despite the lack of reliability, the difference might suggest that some people do, on occasion, interpret omissive causes as non-events that have no temporal anchor. But, the vast majority of participants’ responses suggest that people typically interpret both omissive and orthodox causal relations to yield distinct temporal inferences, in line with prediction 2.

Perhaps the responses participants made in Experiments 1 and 2 reflect egregious errors in reasoning. Consider statement (6), from the Introduction, concerning a student’s failure to do his homework:

6. Not doing his homework caused him to fail.

Prominent philosophers argue that omissions are non-events that do not occur in space or time (Clarke, 2014, p. 38 et seq.). The view is not meant to describe human psychology, but rather to describe metaphysics. And consensus about metaphysics can outline normative, ideal performance. If the previous scholars are right, then people are mistaken whenever they construe non-events as occurring in any location or point in time, or even in a relative place or timepoint. There may be some credence to their view; after all, the following question seems bizarre:

14. Q: # *Where* did he not do his homework?

As Experiments 1 and 2 suggest, however, this question, and its answer, seem sensible:

15. Q: *When* did he not do his homework?
A: He didn’t do his homework before his grade fell.

How can (15) make sense when (14) doesn’t? Two explanations seem viable: either non-events don’t occur in space or time, in which case reasoners err whenever they draw temporal inferences from causal assertions; or else non-events can occur in a temporal context without occurring in a spatial context – a result that undermines theories based on physics simulations. And certain sorts of omissive events may promote temporal inferences more than others. Future research should adjudicate the two proposals.

As Table 1 shows, the results of Experiments 1 and 2 contravene the view that omissions are forces – the force theory may refer to components of vectors as “end points,” but it has no mechanism for inferring temporal relations. The theory could be modified, as any theory can: proponents of the force theory could posit additional processes that keep track of separate forces before they’re combined into a single vector; but such addendums would be akin to Ptolemy’s epicycles, i.e., they would be external to the theory itself, and it’s not clear that they would serve any purpose beyond accounting for the effects we outline.

No studies have directly tested between the contrast view and the model theory, but predictions 3 and 4 distinguish the two (see Table 1). To test them, we carried out an experiment in which participants considered the various possibilities consistent with causes and enabling conditions. If people consider contrasts by default, then they should spontaneously describe contrasting possibilities at least as often as non-contrasting possibilities (prediction 3), and they should describe at most two possibilities (prediction 4).

## Experiment 3

Experiment 3 tested the hypothesis that people represent contrasts by default (Table 1, prediction 3). It elicited natural responses to the different possibilities for orthodox and omissive cause and enabling conditions. Participants read a single short premise, such as:

The absence of a particular preservative causes a substance to decay.

Then they listed the possibilities that corresponded to the premise using an interface designed to help them consider four relevant situations:

The preservative is absent and the substance decays. (not-A & B)
The preservative is present and the substance does not decay. (A & not-B)
The preservative is present and the substance decays. (A & B)
The preservative is absent and the substance does not decay. (not-A & not-B)

We analyzed the order in which participants listed each possibility, as well as the first possibility they listed. The contrast theory predicts that reasoners should list the possibilities that correspond to *not-A* & *B* and *A* & *not-B* equally often when they interpret omissive causation. The model theory predicts that reasoners should list the possibilities in a piecemeal fashion: for omissive causal relations, they should consider the possibility that corresponds to *not-A* & *B* first, then (if at all) the possibility that corresponds to *A* & *not-B*, and finally (if at all) the possibility that corresponds to *A* & *B*. And the theory predicts an analogous trend in latencies: reasoners should consider *not-A* & *B* faster than *A* & *not-B*, and they should consider *A* & *not-B* faster than *A* & *B.* For orthodox causes, the model theory predicts that participants should list the order of possibilities as follows: *A* & *B* > *not-A* & *not-B* > *not-A* & *B*, where ‘ > ’ denotes that the possibility should be listed faster and more often. For enabling conditions, they should consider additional possibilities, i.e., they should be more likely to list *A* & *not-B* for orthodox enablers than orthodox causes, and they should be more likely to list *not-A* & *not-B* for omissive enablers than omissive causes (see Khemlani et al., 2018b; Table 1).

### Method

*Participants.* 31 participants volunteered through the Amazon Mechanical Turk online platform. 22 participants reported no formal logic or advanced mathematical training and the remaining reported introductory to advanced training in logic. All were native English speakers.

*Design, procedure, and materials.* Participants completed 2 practice problems and 8 experimental problems, and they acted as their own controls. Each problem presented a premise that consisted of two events and a causal verb. The experiment manipulated whether the first event concerned orthodox or omissive causation: half the problems used the word “presence” and the other half used the word “absence” when describing the first event. The experiment also manipulated the relevant causal relation: half the problems concerned causation and half concerned enabling conditions. An example problem is as follows:

Suppose the following statement is true:
The [presence/absence] of a particular preservative [causes/enables] a substance to decay.
What is possible given the above statement?

**FIGURE 3:**
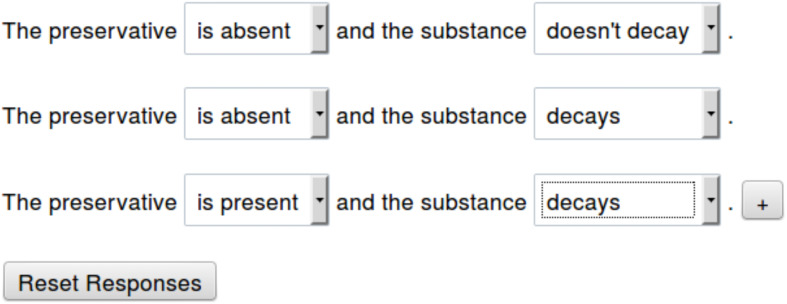
The interface used to elicit responses in Experiment 3. Participants completed sentences using drop-down menus and added possibilities using a button marked “+.”

Participants were then asked to construct a list of possibilities using pre-populated drop-down menus. Figure 3 shows an example of the interface used in Experiment 3. Participants could choose any combination of the possibilities from the drop-down menus, they could change their answer choices at will, and they could add additional sentences if they thought the statement was true in a number of possibilities. The interface allowed the construction of at most four different sentences. The presentation order of the trials was randomized. The order in which participants endorsed possibilities was recorded, as was the latency when the premises appeared, when each item in the list was generated, and when participants pushed a button to finish the trial.

### Results and Discussion

Table 2 shows the percentage of trials on which participants listed the four possible sentences as a function of whether the premise in the trial concerned an orthodox or an omissive causal relation. The table also shows, in parentheses, the percentages of trials on which a given sentence appeared first in the set of sentences listed by the participants. Table 3 shows the mean latencies for such responses after eliminating outliers. We examine possibility listing behavior and their corresponding latencies separately, and report statistical analyses on only those planned comparisons that help adjudicate prediction 3.

**TABLE 2:** Percentages of trials on which participants in Experiment 3 listed four separate possibilities for trials that concerned omissive and orthodox causal relations.

	The four possibilities participants could list			
	
	A & B	A & not-B	not-A & B	not-A & not-B
**Causes**				
Orthodox	**100 (100)**	6 (0)	31 (0)	74 (0)
Omissive	47 (26)	69 (26)	**85 (48)**	19 (0)
**Enables**				
Orthodox	**98 (97)**	27 (2)	27 (2)	73 (0)
Omissive	34 (16)	73 (23)	**87 (58)**	32 (3)

*Bolded cells highlight possibilities that correspond to initial models (see Khemlani et al., 2018b). Parentheses indicate percentages of trials on which participants constructed the corresponding possibility first.*

**TABLE 3:** Mean latencies (in s) to construct each of four separate possibilities in Experiment 3 as a function the causal relation (causes vs. enables) in each trial and as a function of whether the premise described an omissive or an orthodox antecedent.

	The four possibilities participants could list			
	
	A & B	A & not-B	not-A & B	not-A & not-B
**Causes**				
Orthodox	**10.24**	17.49	16.75	15.40
Omissive	15.68	18.45	**14.61**	26.31
**Enables**				
Orthodox	**10.91**	15.09	17.97	19.47
Omissive	16.28	19.53	**14.23**	19.14

*Bolded cells highlight possibilities that correspond to initial models (see Khemlani et al., 2018b).*

*Listing possibilities.* Descriptive statistics revealed that on 26% of responses, participants listed 1 possibility; on 35% of responses, they listed 2 possibilities; on 31% of responses they listed 3 possibilities; and on 8% of responses, they listed all 4 possibilities. These results corroborate prediction 3.

We further analyzed the possibilities people produced to assess the fine-grained predictions of the model theory. A GLMM regression would not suffice to analyze the data because of the multivariate nature of the experimental design, and so we opted to subject the data to a series of non-parametric analyses. For omissive causes, participants listed *not-A* & *B* more often than *A* & *not-B* (85 vs. 69%, respectively; Wilcoxon test, *z* = 2.88, *p* = 0.003, Cliff’s δ = 0.16) and for orthodox causes, they listed *A* & *B* more often than *not-A* & *not-B* (100 vs. 74%, respectively; Wilcoxon test, *z* = 4.00, *p* < 0.0001, Cliff’s δ = 0.26). Both results contravene prediction 3, which states that contrasting possibilities should be listed just as often. The data corroborated the trend predicted by the model theory for omissive causes: participants constructed *not-A* & *B* most often (85% of trials), then *A* & *not-B* (69%), then *A* & *B* (47%), and rarely *not-A* & *not-B* (19%). A non-parametric trend test revealed a significant trend in their responses (Page’s trend test, *z* = 5.16, *p* < 0.0001). A similar trend occurred for orthodox causes, i.e., participants listed *A* & *B* most often (100%), then *not-A* & *not-B* (74%), then *not-A* & *B* (31%), and rarely A & not-B (6%; Page’s trend test, *z* = 6.94, *p* < 0.0001). For omissive enabling conditions, participants likewise listed the possibility corresponding to the initial model, *not-A* & *B*, more often than a contrasting possibility, *A* & *not-B* (87% vs. 73%; Wilcoxon test, *z* = 2.18, *p* = 0.03, Cliff’s δ = 0.14); a similar pattern held for orthodox enabling conditions (*A* & *B*: 97% vs. *not-A* & *not-B*: 73%; Wilcoxon test, *z* = 4.00, *p* < 0.0001, Cliff’s δ = 0.26), and both results violated prediction 3.

One way of understanding participants’ performance is to examine only the first possibility in the set of possibilities they listed: doing so provides insight on their online preferences for possibilities. Participants constructed *not-A* & *B* as a first sentence more often than *A* & *not-B* for omissive causes (48 vs. 26%, respectively; Wilcoxon test, *z* = 2.06, *p* = 0.04; Cliff’s δ = 0.23) and omissive enabling conditions (58 vs. 23%, respectively; Wilcoxon test, *z* = 3.11, *p* = 0.002; Cliff’s δ = 0.35). And they constructed *A* & *B* as a first sentence more often than *not-A* & *not-B* for orthodox causes (100 vs. 0%, respectively) and enabling conditions (97% vs. 0%, respectively). These results would not hold if people simulated contrasts by default.

As Table 2 shows, on 47% of the trials, participants listed the *A* & *B* possibility; they did so more than a chance probability of 25% (Wilcoxon test, *z* = 3.01, *p* = 0.002, Cliff’s δ = 0.06). This possibility does not correspond to a contrast (see Stephan et al., 2017), and so the contrast theory cannot account for it (see Table 1, prediction 4).

*Latencies.* We recorded how long it took participants to list each possibility (see Table 3). The experiment recorded the temporal interval between when the premises were displayed and when people pressed the ‘ + ’ button for the first time, i.e., it recorded how long it took participants to infer an initial possibility. For all subsequent possibilities, it recorded the interval between presses of the ‘ + ’ button; and for the last possibility, it recorded the interval between the ‘ + ’ button and a separate button that participants pressed to indicate that they had finished a trial. Outlier rejection methodology followed the best practices identified by Bakker and Wicherts (2014). Approximately 2.3% of the data were removed as outliers using a threshold of 3^∗^IQR. Data were subjected to non-parametric analyses, which allow latencies to be analyzed without additional transformation. We report planned comparisons for only those latencies most pertinent to testing prediction 3. Participants were faster to list *not-A* & *B* than *A* & *not-B* for both omissive causes (14.61 s vs. 18.45 s; Mann-Whitney test, *z* = 1.76, *p* = 0.08, Cliff’s δ = 0.21) and omissive enabling conditions (14.23 s vs.19.53 s; Mann-Whitney test, *z* = 2.94, *p* = 0.003, Cliff’s δ = 0.34). Likewise, they were faster to list *A* & *B* than *not-A* & *not-B* for both orthodox causes (10.24 s vs. 15.40 s; Mann-Whitney test, *z* = 5.34, *p* < 0.0001, Cliff’s δ = 0.61) and orthodox enabling conditions (10.91 s vs. 19.47 s; Mann-Whitney test, *z* = 5.67, *p* < 0.0001, Cliff’s δ = 0.65). These results further contravene prediction 3, i.e., the notion that people represent contrasts by default. A non-parametric trend test revealed that participants’ latencies for omissive causes marginally followed the trend predicted by the model theory (i.e., *not-A* & *B* < *A* & *not-B* < *A* & *B* < *not-A* & *not-B*; Jonckheere’s trend test, *z* = 1.52, *p* = 0.06). A similar analysis reliably yielded the predicted trend for orthodox causes (i.e., *A* & *B* < *not-A* & *not-B* < *not-A* & *B* < *A* & *not-B*; Jonckheere’s trend test, *z* = 3.03, *p* = 0.001).

In sum, the results of Experiment 3 suggest that reasoners tend to consider those possibilities that correspond to initial models before all other possibilities. When they think about other possibilities, they tend to consider them in a piecemeal fashion. The trends in the possibilities people tend to list and their corresponding latencies suggest that people consider possibilities one after another in a pattern uniquely predicted by the model theory. The results are incompatible with the contrast theory, which argues that people represent contrasting possibilities by default, and that they consider two possibilities at most for omissive causes. The results show that they consider three possibilities, in accordance with the model theory.

The force theory cannot explain the patterns found in Experiment 3, either: while the theory does not predict that people should represent contrasting possibilities by default (see Table 1), it also has no mechanism to account for why people privilege some possibilities over others, or how they could consider multiple possibilities in the trends observed.

## General Discussion

Omissive causes have challenged metaphysicians’ views of causation for decades. How can nothing – an “omission,” i.e., the failure of a particular event to transpire – cause something? Psychologists concur that omissions have representational content, and recent theorists concur that people mentally simulate the world when they reason. But three proposals of omissive causation disagree on the structure of those simulations.

One theory argues that causes are akin to the transmission of a quantity, i.e., a force (Wolff et al., 2010). It posits that omissive causes are equivalent to double preventions: an event (e.g., eating lunch) would otherwise prevent some outcome (e.g., getting hungry), but some other event – say, a busy schedule – prevents the first event from happening. The proposal is plausible; its formalism is not. The theory argues that people represent causes, enabling conditions, and prevention as distinct arrangements of force vectors. Force vectors represent only the directions and magnitudes of forces, and that information is sufficient for a mathematical formalism to explain how such forces combine. But it ignores a central element of causal reasoning: time. Hence, the force theory cannot explain how people draw temporal conclusions from causal assertions.

People typically reason that outcomes cannot precede their causes. The assumption is sensible in the case of “orthodox” causes, as in the following:

16. Overfishing caused the algal bloom.

It seems obvious that overfishing occurred first, the algal bloom afterward. But an analogous temporal inference is troubling in the case of omissive causes, as in:

17. A lack of predatory fish caused the algal bloom.

Presumably, the lack of such fish occurred both before, during, and after the algal bloom, and so it may seem less sensible to infer that the lack occurred only before the bloom. But Experiments 1 and 2 show that reasoners do so: they considered *the lack of A caused B* to imply that *the lack of A happened before B*, but not *the lack of A happened after B.* This systematicity cannot be accounted for if the mind represents omissive causes as force vectors.

A second theory of the representation of omissions argues that (17) is best construed by simulating a relevant counterfactual, i.e., the situation in which predatory fish were abundant and algae never flourished (Stephan et al., 2017). The counterfactual serves as a contrasting possibility, and the theory was inspired by similar arguments by philosophers (e.g., Schaffer, 2005; Bernstein, 2014). It argues that reasoners base judgments on such contrasts as well as on the situations they contrast against. Hence, people should represent two different scenarios in mind by default. The proposal is warranted by studies that show that people are capable of representing two possibilities when they reason about counterfactuals (Byrne, 2005). And so we conducted a study to examine whether people represent omissive causes as contrasting possibilities. Experiment 3 gave participants descriptions of omissive causes (as well as omissive enabling conditions, orthodox causes, and orthodox enabling conditions) and asked them to list what was possible given the truth of the statement. The patterns of the participants’ responses suggested that they did not consider contrasting alternatives by default. Instead, for each type of statement, they privileged one possibility over all others. They listed that possibility first, most often, and fastest.

The results of Experiments 1, 2, and 3 cannot be explained by the preceding theories. But a third theory can account for them. The theory posits that people represent omissive causes as temporally ordered sets of possibilities – i.e., models (Khemlani et al., 2018b). Hence, reasoners typically represent (17) as an initial model, i.e., a single iconic possibility that renders the premise true. The initial model can be depicted in the following diagram:

¬ predators   algal-bloom

The temporal ordering of the possibility allows it to be scanned from left to right to yield inferences about the chronology of events. Hence, the model theory predicts the systematic temporal inferences that reasoners made in Experiments 1 and 2.

In most cases, the initial model of an omissive cause suffices. But in some cases, reasoners have to consider alternative possibilities. When they think about causes that occurred in the past, they may consider counterfactual alternatives. When they think about future causal relations, they may think about alternative outcomes. In either case, the simulation of alternative possibilities requires effort – and so people should consider alternative possibilities less often and more slowly than they consider possibilities that correspond to the initial model. The results of Experiment 3 bear out these predictions. For omissive causes, reasoners listed the initial model above more frequently than any other possibility. But they also listed two other possibilities more often than not, i.e., those in which the cause occurred and the effect did not, and those in which the cause occurred and the effect occurred for some other reason. For (17), those possibilities are as follows:

predators     ¬ algal-bloom
predators        algal-bloom

Participants’ responses likewise validated the theory’s predictions for the difference between causes and enabling conditions and for the difference between omissive and orthodox causes.

Theories of causal reasoning must explain how people treat absences and failures as agents of causation. A unified approach is needed, one whose central mechanisms apply to orthodox and omissive causes alike. Of the three theories discussed in this paper – the force theory, the contrast theory, and the model theory – only the model theory bases omissive causal reasoning on similar processes as orthodox causal reasoning. The force theory posits that reasoners understand omissive causes, such as the one in (17), by interpreting them as double preventions, i.e., situations in which some unknown cause prevents another cause (e.g., the presence of predatory fish) from preventing an effect (e.g., an algal bloom). But, the force theory does not appeal to double preventions to explain how people understand orthodox causes – and so the theory uses different mechanisms for orthodox and omissive causation. The contrast theory argues that people reason about causation by considering contrasting possibilities: to understand the orthodox causation in (16), reasoners consider what would’ve happened if overfishing hadn’t occurred, i.e., they replace something that did happen with something that didn’t. The opposite approach is infeasible for omissive causation: you cannot replace what didn’t happen with something that did happen, because many possible things could have happened. So, the contrast theory appeals to running simulations in order to ascertain what would have likely happened in place of the absence. This approach, too, adopts a novel mechanism to cope with reasoning about omissions. The model theory, however, does not: reasoners construct models of omissive causes just as they do models of orthodox causes. They tend to prefer one mental model to describe a cause, whether omissive or orthodox, and they deliberate in order to consider alternative possibilities. These processes suffice to explain the phenomena most relevant to understanding omissive causation (see Table 1), and they minimally extend existing theoretical proposals. Hence, the model theory provides the most conservative approach to explaining phenomena relevant to omissive causation.

People’s intuitions about causation matter, not just to psychologists interested in understanding causal reasoning. They matter to AI researchers, who wish to develop systems that interact with humans to identify and reason about causality. Many AI systems mimic learning processes, i.e., they scour massive amounts of data to recognize associative patterns between recognizable events. Many such machine learning algorithms seem well suited for finding causal patterns for orthodox events, i.e., events that can be identified – but the concept of omissive causation challenges such algorithms, because learning algorithms cannot associate an absence of data with the presence of some outcome (see, e.g., Van Hamme and Wasserman, 1994). For example, no matter how many times a machine learning algorithm experiences instances of cellphone batteries dying, it would have difficulty learning that not charging a cellphone causes it to die. Hence, new computational techniques are necessary to capture efficient ways of computing omissive causation.

Recent computational and theoretical approaches posit that all human reasoning is inherently “modal,” i.e., it is based on the consideration of possibilities (Johnson-Laird et al., 2015; Phillips et al., 2019). In the case of omissive causation, modal cognition helps solve a long-standing mystery of how the mind represents omissions: it does so by representing the possibilities to which omissions refer. The present results suggest that those possibilities are iconic in nature, and that reasoners tend to privilege one possibility over others. The fact that reasoners can represent an omissive cause with one possibility may explain why reasoning about omissions is not particularly difficult in daily life – it is easy to understand, for instance, that not charging your cellphone *caused* its battery to die. But considering just one possibility may not be enough in order to make accurate inferences. Hence, reasoners who deliberate and consider alternative possibilities may make fewer mistakes. At present, the only account capable of explaining mistakes – as well as optimal performance – in reasoning about omissive causation is the model theory.

## Data Availability Statement

The datasets presented in this study can be found in online repositories. The names of the repository/repositories and accession number(s) can be found in the article/supplementary material.

## Ethics Statement

The studies involving human participants were reviewed and approved by Naval Research Laboratory Institutional Review Board. The patients/participants provided their written informed consent to participate in this study.

## Author Contributions

SK contributed to developing the theory and writing the article. PB contributed to developing the theory. GB and HH contributed to developing the theory, writing the article, running the experiments, analyzing the data, and preparing the tables. CW contributed to writing the article. All authors contributed to the article and approved the submitted version.

## Conflict of Interest

The authors declare that the research was conducted in the absence of any commercial or financial relationships that could be construed as a potential conflict of interest.
